# Global research and learning agenda for building evidence on contraceptive-induced menstrual changes for research, product development, policies, and programs

**DOI:** 10.12688/gatesopenres.13609.1

**Published:** 2022-04-19

**Authors:** Emily Hoppes, Chukwuemeka Nwachukwu, Julie Hennegan, Diana L. Blithe, Amanda Cordova-Gomez, Hilary Critchley, Gustavo F. Doncel, Laneta J. Dorflinger, Lisa B. Haddad, Amelia C.L. Mackenzie, Jacqueline A. Maybin, Kelle Moley, Kavita Nanda, Carolina Sales Vieira, Bellington Vwalika, Simon P.S. Kibira, Alexandria Mickler, Funmilola M. OlaOlorun, Chelsea B. Polis, Marni Sommer, Katie M. Williams, Eva Lathrop, Tanya Mahajan, Kate H. Rademacher, Marsden Solomon, Katrina Wilson, Lucy C. Wilson, Lillian Rountree

**Affiliations:** 1 FHI 360, Durham, NC, 27701, USA; 2 United States Agency for International Development (USAID), Washington, District of Columbia, 20523, USA; 3 Maternal Child and Adolescent Health Program, Burnet Institute, Melbourne, VIC 3004, Australia; 4 Contraceptive Development Program, Eunice Kennedy Shriver National Institute of Child Health and Human Development, National Institutes of Health, Bethesda, Maryland, 20892, USA; 5 MRC Centre for Reproductive Health, Queen’s Medical Research Institute, University of Edinburgh, Edinburgh, EH8 9YL, UK; 6 CONRAD, Eastern Virginia Medical School, Norfolk, Virginia, 23507, USA; 7 Center for Biomedical Research, Population Council, New York, New York, 10017, USA; 8 Bill & Melinda Gates Foundation, Seattle, Washington, 98109, USA; 9 Department of Gynecology and Obstetrics, Ribeirao Preto Medical School, University of São Paulo, São Paulo, Brazil; 10 Departments of Obstetrics and Gynaecology, University of Zambia School of Medicine, Lusaka, Zambia; 11 Department of Community Health and Behavioural Sciences, School of Public Health, Makerere University, Kampala, Uganda; 12 Department of Community Medicine, University of Ibadan, Ibadan, Nigeria; 13 Independent Researcher, Brooklyn, New York, USA; 14 Department of Sociomedical Sciences, Mailman School of Public Health, Columbia University, New York, New York, 10032, USA; 15 Population Services International, Washington, District of Columbia, 20526, USA; 16 The Pad Project, New Delhi, India; 17 MSI Reproductive Choices, London, W1T 6LP, UK; 18 Rising Outcomes, Hillsborough, NC, 27278, USA

**Keywords:** family planning, contraceptive, menstrual health, contraceptive-induced menstrual change, CIMC, menstrual change, research agenda, research and learning agenda

## Abstract

**Background**: Contraceptive-induced menstrual changes (CIMCs) can affect family planning (FP) users’ lives in both positive and negative ways, resulting in both opportunities and consequences. Despite this, and despite the important links between FP and menstrual health (MH), neither field adequately addresses CIMCs, including in research, product development, policies, and programs globally.

**Methods**: In November 2020, a convening of both MH and FP experts reviewed the existing evidence on CIMCs and identified significant gaps in key areas.

**Results**: These gaps led to the establishment of a CIMC Task Force in April 2021 and the development of the *Global Research and Learning Agenda: Building Evidence on Contraceptive-Induced Menstrual Changes in Research, Product Development, Policies, and Programs Globally *(the CIMC RLA) ***, *** which includes four research agendas for (1) measurement, (2) contraceptive research and development (R&D) and biomedical research, (3) social-behavioral and user preferences research, and (4) programmatic research.

**Conclusions**: Guided by the CIMC RLA, researchers, product developers, health care providers, program implementers, advocates, policymakers, and funders are urged to conduct research and implement strategies to address the beneficial and negative effects of CIMCs and support the integration of FP and MH. CIMCs need to be addressed to improve the health and well-being of women, girls, and other people who menstruate and use contraceptives globally.

***Disclaimer*** *: The views expressed in this article are those of the authors. Publication in Gates Open Research does not imply endorsement by the Gates Foundation.*

## Introduction

Contraceptive-induced menstrual changes (CIMCs) affect contraceptive users’ lives in both positive and negative ways. These include consequences such as dissatisfaction with and discontinuation of contraceptives, as well as opportunities ^1^, such as improved quality of life and potential treatment of menstrual disorders ^2^. Despite the important links between family planning (FP) and menstrual health (MH), ^a^ neither field adequately addresses CIMCs, including in research, product development, policies, and programs globally.

## Contraceptive-induced menstrual changes

CIMCs encompass all changes to a users’ menstrual cycle caused by using contraception, including:

- Changes in bleeding duration, volume, frequency, and/or regularity/predictability
- Changes in blood (and other uterine and cervical effluent) consistency, color, and/or smell
- Changes in uterine cramping and pain
- Changes in other symptoms before, during, and after menstruation (e.g., migraines, breast tenderness, gastrointestinal symptoms)
- Changes in experiences of menstrual and gynecologic disorders and symptoms ^b^
- Changes over time with continued contraceptive method use
- Short-term changes to the menstrual cycle after contraceptive discontinuation

Some individuals dislike CIMCs, which can contribute to dissatisfaction or discontinuation or non-use of contraception ^1, 3– 5^. These negative reactions are the result of the varied and real impacts of CIMCs on users’ lives and their beliefs surrounding menstruation. CIMCs, particularly heavier, longer, irregular, or painful bleeding, may exacerbate difficulties in managing menstruation, including changes in the quantity or type of menstrual materials needed, increased need for analgesics, and an increase in the need for safe, private, accessible water, sanitation, and hygiene (WASH) facilities ^6, 7^. In addition, CIMCs can have significant effects on users’ abilities to participate in regular activities like school, work, sex, and social and religious activities ^4, 8^. For example, in some contexts, social norms inhibit users from participating in religious practices or household work like cooking when they are menstruating ^10^. CIMCs can also be associated with psychosocial impacts caused by the stress of managing these changes and worry related to hiding CIMCs among those trying to use their contraceptive method discreetly ^11^. CIMCs can also negatively affect sexual satisfaction and well-being ^12^. In addition, beliefs about CIMCs can reduce individuals’ motivation to begin or continue using contraception, and can influence the attitudes and behaviors of providers ^13^. Some contraceptive users fear that CIMCs indicate, or can lead to, negative health consequences, especially bleeding that is heavier in volume or longer in duration. On the other hand, some users may fear that contraceptive-induced amenorrhea—or paused bleeding—means there is a buildup of “dirty” or “bad” blood in their bodies that might indicate or lead to major health issues including infertility, although these are not clinically documented health effects of contraceptive-induced amenorrhea ^4, 14– 16^.

CIMCs can also have advantages that motivate individuals to begin and/or continue contraceptive use. Reduced menstrual bleeding, pain, or cramping, as well as paused bleeding can offer increased freedom to engage in regular activities, improved convenience, improved sexual satisfaction, decreased stress and worry, and reduced spending if fewer menstrual materials are needed ^2, 17^. Some individuals choose to use contraception primarily, or at least in part, for the resulting beneficial menstrual changes, including the management of menstrual and gynecologic disorders and symptoms, such as heavy menstrual bleeding, which affects approximately 30 percent of those who menstruate ^18^, and endometriosis, which affects an estimated 10% of menstruators worldwide ^19^. Contraceptives that reduce bleeding may also prevent or improve other health conditions, including iron deficiency and iron deficiency anemia, which can be caused by heavy menstrual bleeding and affects about a third of women of reproductive age globally ^20^. Finally, CIMCs can be beneficial for transgender and gender expansive persons who may use contraceptives to induce amenorrhea and reduce the effects menstruation may have on gender dysphoria ^21^.

### Evidence and knowledge gaps

In November 2020, a convening of both MH and FP experts reviewed the existing evidence on CIMCs and identified significant gaps in key areas ^22^. Critically, not enough is known about the biological mechanisms that underlie CIMCs; therefore, therapies for preventing undesired CIMCs and for prolonging desired CIMCs lack a robust mechanistic foundation ^23^. This lack of mechanistic knowledge impacts the potential for research and development (R&D) to lead to new and innovative contraceptives that might also be treatments for menstrual and gynecologic disorders and symptoms ^24^. While evidence exists around the preferences of contraceptive users related to CIMCs, not enough is known about the social and relational influences that shape these preferences and existing evidence is from a limited population that lacks diversity. More research is needed to understand the full impact of CIMCs on contraceptive use, menstrual health, and quality of life ^4, 25^. There is also a substantial evidence gap in understanding the most effective programs and interventions to address CIMCs, including ideal approaches for counseling and the potential impact of integrating FP services and MH services ^c^ ^26, 27 ^. Finally, a lack of standardized and validated measures for different aspects of CIMCs and harmonization across the measurement of biological mechanisms, user preferences, social influences, impacts, and programs compounds the evidence gap ^22^.

These gaps led to the establishment of a CIMC Task Force in April 2021 and the development of the ***Global Research and Learning Agenda: Building Evidence on Contraceptive-Induced Menstrual Changes in Research, Product Development, Policies, and Programs Globally,*** referred to below as the “CIMC RLA” ^28^.

## The contraceptive-induced menstrual changes global research and learning agenda

The CIMC RLA includes four research agendas focused on: (1) measurement, (2) contraceptive research and development (R&D) and biomedical research, (3) social-behavioral and user preferences research, and (4) programmatic research. It was developed to provide guidance to researchers, product developers, health care providers, program implementers, advocates, policymakers, and funders interested in expanding understanding of CIMCs. For all four agendas, it is essential that research is conducted with diverse populations across different locations, races and ethnicities, socio-economic status, ages, abilities/disabilities, sexual orientations, and gender identities, and that researchers recognize the complexity and intersection of identities that play a role in people’s perceptions, experiences, and behavior. In addition, groups who have been historically systematically marginalized or underserved should be involved to the extent possible in this research, including youth, perimenopausal people, people with disabilities, people living with HIV, postpartum people, refugees, migrants or other mobile populations, sex workers, people in the LGBTQ (lesbian, gay, bisexual, transgender, queer) community, survivors of abuse and violence, and those who are incarcerated.

### Measurement research agenda

**Figure 1.**
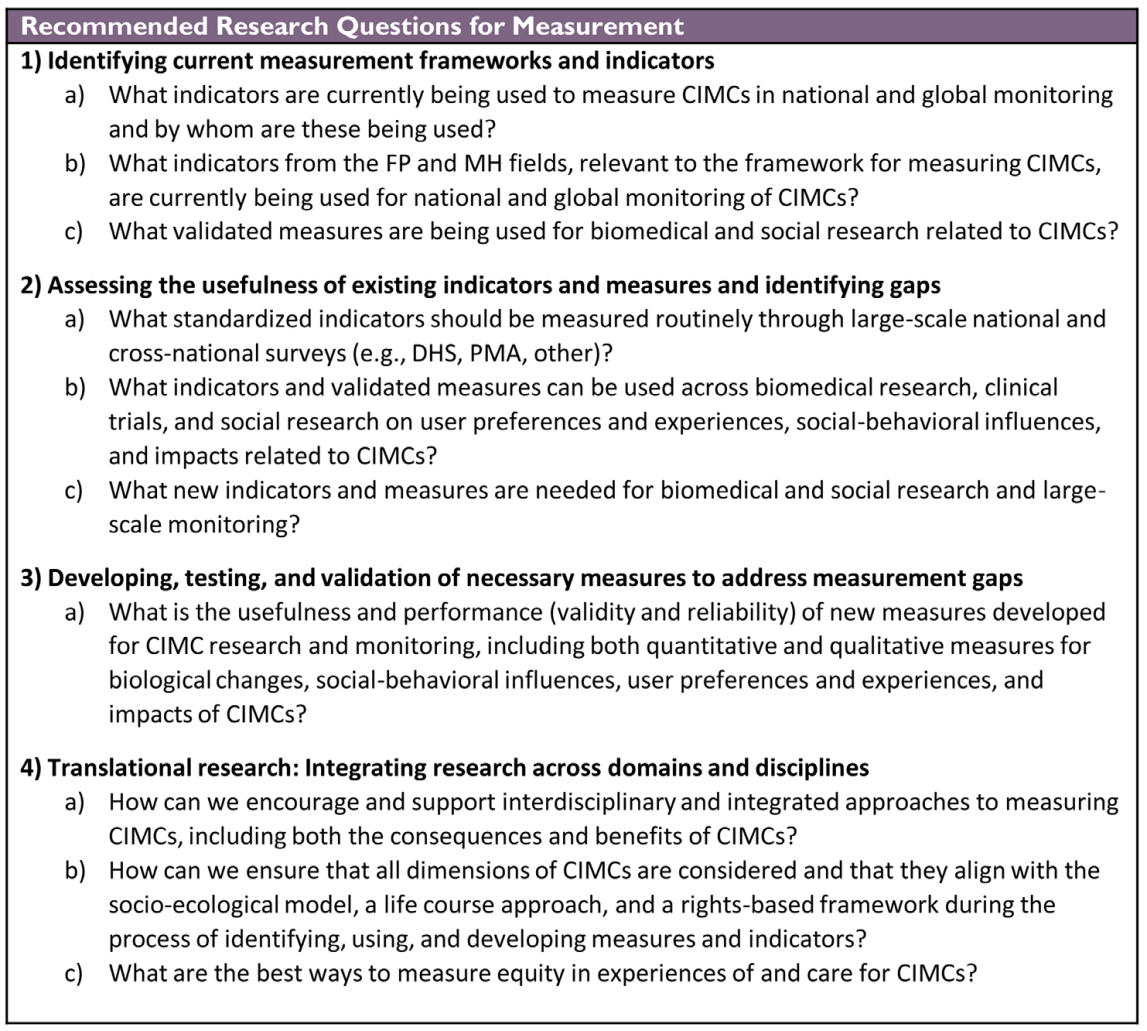
Measurement Research Agenda for Contraceptive-Induced Menstrual Changes.

**Figure 2.**
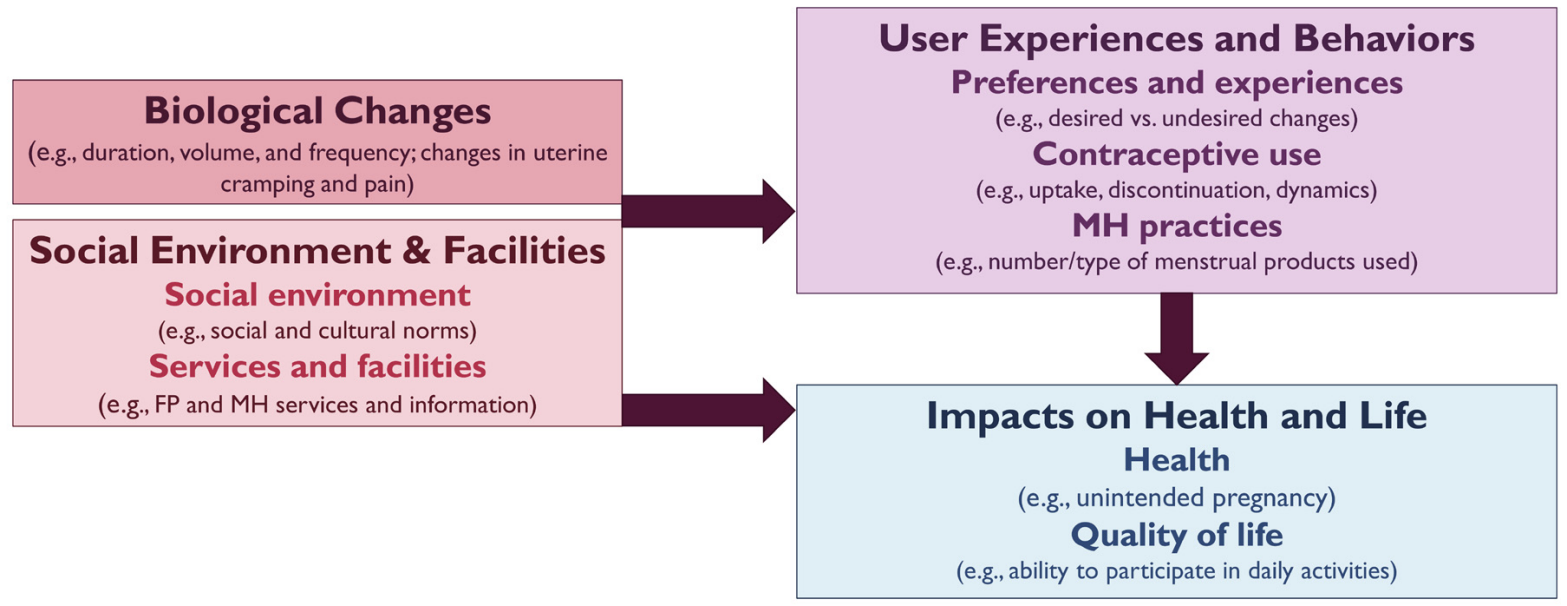
Contraceptive-Induced Menstrual Changes Measurement Framework.

Across research efforts, the measurements used shape what is learned. For CIMCs, an integrated and interdisciplinary approach is needed to ensure essential concepts are identified and measured appropriately. Figure 1 provides the full measurement research agenda. Future CIMC research and programs should be informed by a harmonized measurement framework that includes indicators related to biological changes, social environments, facilities and services, user experiences, preferences, and behaviors, and impacts on health and life ( Figure 2). As a priority, those working in CIMC research should review the indicators and tools being used across disciplines to identify opportunities for standardization and gaps to be addressed. 

### Contraceptive R&D and biomedical research agenda

**Figure 3.**
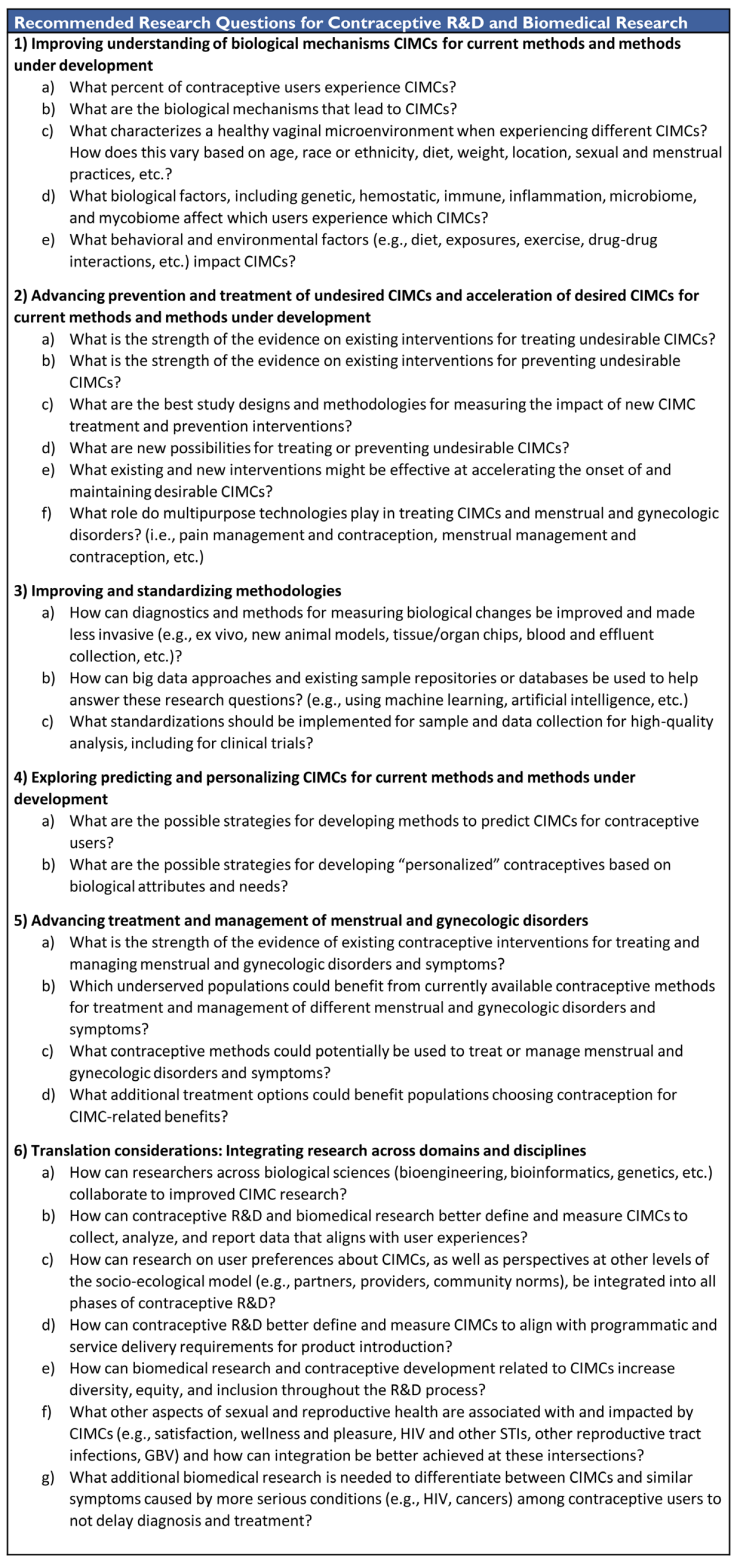
Contraceptive R&D and Biomedical Research Agenda for Contraceptive-Induced Menstrual Changes.

The full agenda for contraceptive R&D and biomedical research is provided in Figure 3. Research in this area should focus on: (1) understanding the biological mechanisms that lead to CIMCs and factors that affect these mechanisms; (2) developing evidence-based prevention and treatment options for undesired CIMCs and options to accelerate and maintain desired CIMCs; and (3) understanding the use of existing and new contraceptive methods to treat menstrual and gynecologic disorders and symptoms. This work should integrate users’ preferences and needs related to CIMCs into product development. As a priority, researchers should work to streamline and improve research definitions, measurement, methodologies, and analyses.

### Social behavioral and user preferences research agenda

**Figure 4.**
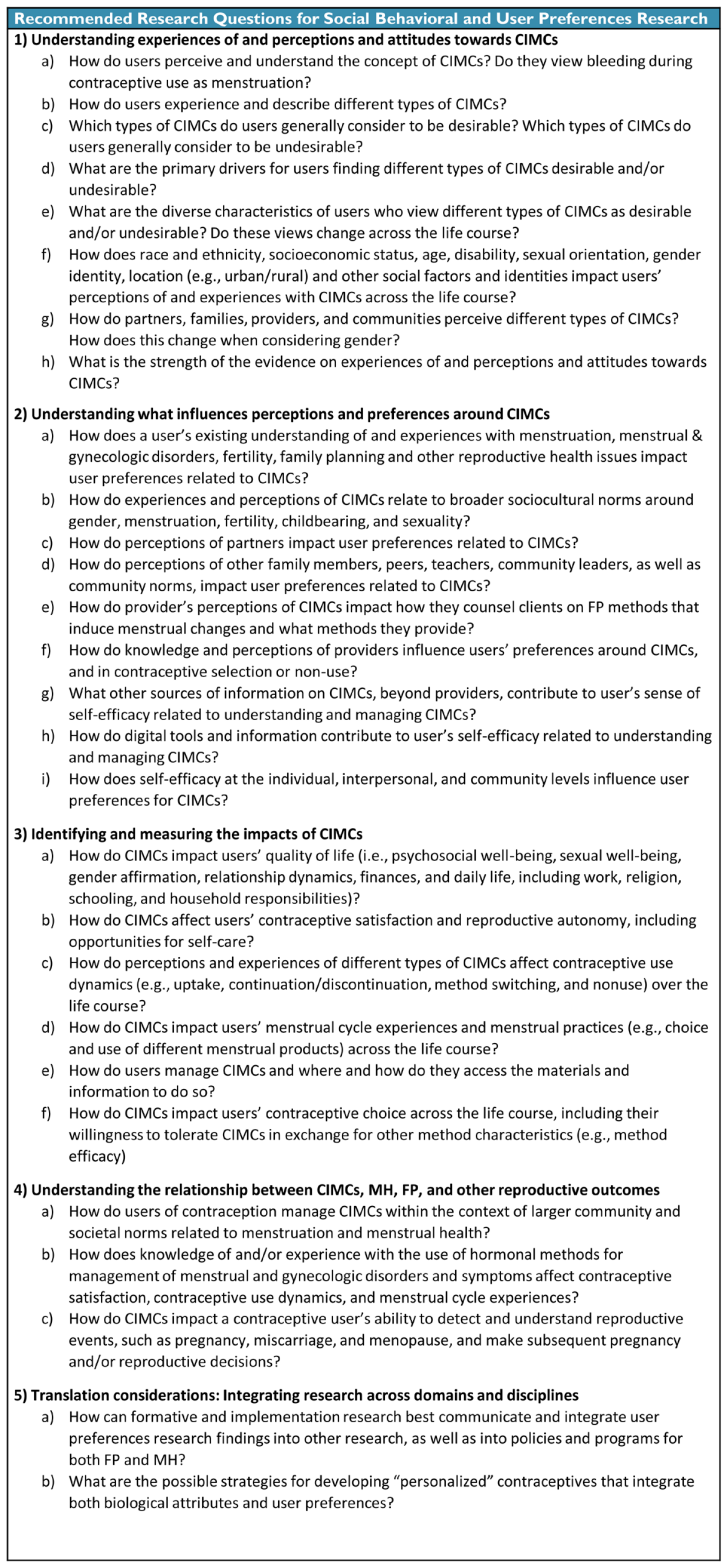
Social Behavioral and User Preferences Research Agenda for Contraceptive-Induced Menstrual Changes.

Figure 4 provides the agenda for better understanding users’ perceptions, attitudes, and experiences related to CIMCs. Future social-behavioral research should seek to understand: (1) the nuance and diversity of perceptions, attitudes, and practices related to all types of CIMCs; (2) the factors that influence CIMC perceptions, attitudes, and practices, including at the individual, interpersonal, and wider socio-ecological levels and across the life course; and (3) the impacts of CIMCs on users’ lives, including their FP and MH practices and decision-making. As a priority, socio-behavioral researchers should assess the state and strength of the existing evidence related to CIMC perceptions.

### Programmatic research agenda

**Figure 5.**
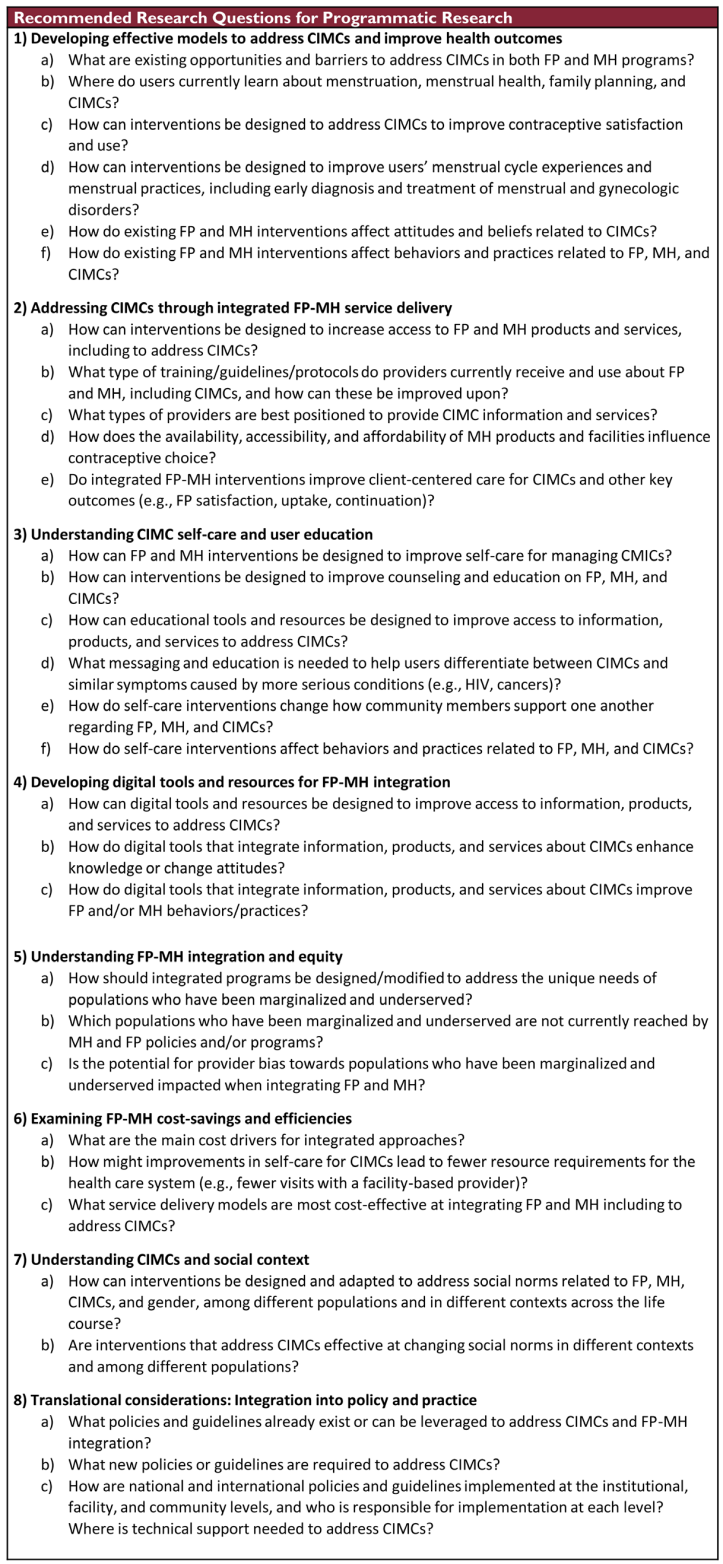
Programmatic Research Agenda for Contraceptive-Induced Menstrual Changes.

When designing and testing ways to address CIMCs through education, counseling, and provision of services, it is important to monitor progress, evaluate impact on a wide variety of measures related to CIMCs, MH, FP, and other areas of sexual and reproductive health and rights (SRHR), and assess the cost-effectiveness of various approaches as well as equity in access. It is also critical to document successes and failures, adjust services accordingly, and disseminate findings to key stakeholders. Key evaluation questions that can be included in implementation science and routine or enhanced monitoring and evaluation are outlined in Figure 5. Future programmatic research should prioritize identifying, defining, and designing how FP and MH can be effectively integrated, including to address CIMCs.

### Cross-cutting considerations and foundations

The CIMC RLA is grounded in the socioecological model ^29^ and a life course approach ^30^. Therefore, we call for research related to CIMCs to: (1) consider the impact of different levels of socio-ecological influence; (2) consider the changing experiences and preferences of users across the reproductive life course, from menarche to menopause; (3) integrate equity using a rights-based framework including considerations for social and environmental determinants of health; and (4) consider and incorporate equity, choice, gender, and self-care.

## Conclusion

Guided by the CIMC RLA, researchers, product developers, health care providers, program implementers, advocates, policymakers, and funders are urged to conduct research and implement strategies to address the beneficial and negative effects of CIMCs and support the integration of FP and MH. Due consideration of CIMCs will help to avoid missed opportunities to integrate MH into sexual and reproductive health and vice versa. Moving forward, CIMCs need to be addressed to improve the health and well-being of women, girls, and other people who menstruate and use contraceptives globally.

## Data availability

No data are associated with this article.
