# Supplementary material for: Global research and learning agenda for building evidence on contraceptive-induced menstrual changes for research, product development, policies, and programs
Source: Gates Open Res. 2022 Apr 19;6:49. [Version 1] doi: 10.12688/gatesopenres.13609.1 (PMC9114387; doi:10.12688/gatesopenres.13609.1)
Supplement: Peer review history

# Peer review: Reviewer response for version 1

**DOI:** 10.21956/gatesopenres.14882.r31980

**Published:** 2022-05-17

**Authors:** Alison B. Edelman

**Affiliations:** 1 Department of Obstetrics and Gynecology, School of Medicine, Oregon Health Science University, Portland, OR, USA

This publication outlines the existing research, research gaps, and strategic planning processes around outcomes, terminology, and future research in the arena of contraceptive-induced menstrual changes. CMIC research needs core outcomes and an updated shared agenda in order to ensure studies both report on clinically relevant outcomes, and meta-analyses can be performed to give more robust health evidence which may result in a definite answer or again, help to better identify the next research steps. The inclusion of patient input regarding these outcomes is also essential to ensuring the research addresses the need of the end user.

 This paper describes the process of how to get there and provides an organized path forward.

 As a frequent peer reviewer, I can honestly say it was a pleasure reading this manuscript – it was easy to read, well-written and thoughtful. It will add significantly to the literature and it will be an incredibly useful reference for those in the field. I have no minor or major edits.

Does the article adequately reference differing views and opinions?

Yes

Are all factual statements correct, and are statements and arguments made adequately supported by citations?

Yes

Is the Open Letter written in accessible language?

Yes

Where applicable, are recommendations and next steps explained clearly for others to follow?

Yes

Is the rationale for the Open Letter provided in sufficient detail?

Yes

Reviewer Expertise:

Routine and complex family planning, contraceptive induced menstrual changes, menstrual health, emergency and post-coital contraception, contraceptive development, contraceptive pharmacodynamics

I confirm that I have read this submission and believe that I have an appropriate level of expertise to confirm that it is of an acceptable scientific standard.

# Peer review: Reviewer response for version 1

**DOI:** 10.21956/gatesopenres.14882.r31981

**Published:** 2022-05-16

**Authors:** Peter Bundi Gichangi

**Affiliations:** 1 International Centre for Reproductive Health-Kenya, Nairobi, Kenya; 2 Technical University of Mombasa, Mombasa, Kenya; 3 Department of Public Health and Primary Care, Faculty of Medicine and Health Sciences, Ghent University, Ghent, Belgium

The topic being addressed is quite important with significant impact on the health of the individuals as well as uptake and discontinuation of family planning/contraceptives (FP/C). This is a topic which has not been addressed adequately in literature.

 The framework proposed to ensure there is research which is guided by identified research questions is likely to result in coherent research output.

 The question I am not sure is addressed adequately is the role of comprehensive counselling as measured using method plus index in Performance Monitoring for Action (PMA) or DHS. This should be considered.

Does the article adequately reference differing views and opinions?

Yes

Are all factual statements correct, and are statements and arguments made adequately supported by citations?

Yes

Is the Open Letter written in accessible language?

Yes

Where applicable, are recommendations and next steps explained clearly for others to follow?

Yes

Is the rationale for the Open Letter provided in sufficient detail?

Yes

Reviewer Expertise:

Sexual and Reproductive Health, Family Planning, gender based violence, cervical cancer.

I confirm that I have read this submission and believe that I have an appropriate level of expertise to confirm that it is of an acceptable scientific standard.
